# 

**Published:** 1825-12

**Authors:** 


### MONTHLY CATALOGUE OF MEDICAL BOOKS.

An Essay on the Nature, Causes, and Treatment, of Water in the Brain. By William Shearman, m.d. Member of the Royal College of Physicians, and senior Physician to the Royal West London Infirmary.—London, 1825.

A Practical Treatise on the Arterial System: intended to illustrate the Importance of studying the Anastomoses, in reference to the Rationale of the new Operation for Aneurism; and the Surgical Treatment of Hemorrhage. With original coloured Plans. By Thomas Turner, Member of the Royal College of Surgeons of London; Lecturer on Anatomy; and Author of "Outlines of a System of Medico-Chirurgical Education," &c. &c. &c.—London, 1825.

Elements of Physiology. By K. A. Rudolphi, m.d. Professor of Medicine, and Member of the Royal Academy of Sciences of Bérlin. Translated from the German, by Wm. Dunbar How, m.d. In three Volumes. Vol. I.—London, 1825.

Additional Observations on the Treatment of certain severe Forms of Hemorrhoidal Excrescence, illustrated by Cases: with the History of a Case in which an enlarged Parotid Gland was successfully removed. By John Kirby, a.b. lately President of the Royal College of Surgeons in Ireland; one of the Surgeons of the Coombe Hospital, and of the Charitable Infirmary, Jervis-street; Lecturer on the Theory and Practice of Surgery at the Anatomical Theatre, Peter-street, Dublin.—Dublin, 1825.

A Manual of Anatomy, arranged so as to afford a concise and accurate Description of the different Parts of the Human Body. From the French of A. L. I. Bayle. Revised and improved by Wm. Bennett, m.d.—Edinburgh, 1825.

Sketches of the most prevalent Diseases of India; comprising a Treatise on the Epidemic Cholera of the East; Statistical and Topographical Reports of the Diseases in the different Divisions of the Army, under the Madras Presidency: embracing also the annual Rate of Mortality, &c. of European Troops, and practical Observations on the Effects of Calomel on the Alimentary Canal, and on the Diseases most prevalent in India. Illustrated by Tables and Plates. By James Annesley, Esq. Madras Medical Establishment; lately in charge of the General Hospital, Madras, and Garrison-Surgeon to Fort St. George.—London, 1825.

On Cholera, more especially as it has occurred during late Years in British India: a Letter addressed to Sir James M'Grigor, m.d. Director-General of the Medical Department of the Army, &c. &c, &c. By Thomas Brown, Surgeon, Musselburgh.—Edinburgh, 1824.

Medical Report of the Fever Hospital and House of Recovery, Cork-street, Dublin, for the Year 1825; with Observations on the Contagion of Plague and Typhus Fever. By Richard Grattan, m.d. a.b. t.c.d. &c.—Dublin, 1825.

A Practical Treatise on Diabetes: with Observations on the Tabes Diuretica, or Urinary Consumption, especially as it occurs in Children; and on Urinary Fluxes in general. With an Appendix of Dissections and Cases, illustrative of a successful Mode of Treatment; and a Postscript of Practical Directions for examining the Urine in these Diseases. By Robert Venables, m.b. Physician to the Henley Dispensary, &c. &c. &c.—London, 1825.

An Introduction to the Use of the Stethoscope; with its Application to the Diagnosis in Diseases of the Thoracic Viscera; including the Pathology of these various Affections. By William Stokes, m.d.—Edinburgh, 1825.

A Short Description of the Bones, together with their several Connexions with each other, and with the Muscles. By John F. South, Lecturer on Anatomy at St. Thomas's Hospital.—London, 1825.

Observations on Tetanus; illustrated by Cases, in which a new and successful Mode of Treatment has been adopted. By Henry Ward, Surgeon.—Gloucester, 1825.

Observations on the Transfusion of Blood; with an Account of two Cases of Uterine Hemorrhage, in which that Operation has been recently performed with Success. By Charles Waller, Member of the Royal College of Surgeons, and of the Hunterian Society; Surgeon to the City of London and Southwark Midwifery Institution, &c.—London, 1825.

### NOTICE TO CORRESPONDENTS.

*The Communications of* Dr. BLAKE, Mr. ASHBURY, Mr. FROST, Dr. SAYER, Mr. CLARK, Mr. RICHMOND, Mr. RICHARDS, Dr. GILBY, *and* Mr. DE BOELTICHER, *have been received.*

*The Letter of* "HUMANUS" *is not fitted for our Journal.*

*The Paper on* Tic Douloureux *cannot be admitted.*

*We will feel much obliged to* Dr. STOKER *for the continuation of his Paper: the part sent will not occupy more than three or four pages.*

*Various Papers, the receipt of which was acknowledged last month, have been postponed for want of room: these in our next.*

*N.B.—Correspondents are particularly requested not to send Pamphlets by post: we cannot receive them.*

### INDEX

OF

THE FIFTY-FOURTH VOLUME

OF THE

### London Medical and Physical Journal.

PAGE

**Abdomen**, wound of the parietes of 466
———, encysted tumors of the 343
Abscess in the Liver, on . . 470
Abscesses in the Kidneys, on . 474
Acid, on the Prussic . . 347
Affections of the Stomach, on . 394
——— Liver, on . 469
——— Kidneys, on . 473
———, diseased, of the Urinary Bladder . . . . 476
——— Womb, on . 478
——— Ovarium, on 479
Alteration in the bones of the head 434
Alterations, morbid, in new-born infants . . . . . 81
Amenorrhœa, on the use of injections in . . . . 434
Analysis of Opium, on the . 111
Aneurismal diathesis, on the . 433
Angina Pectoris, on . . 393
Antimony, tartarized, on the ointment of . . . . 346
Animals, rabid, existence of Lyssæ in . . . . . 251
Antilles, reproduction of the Leech in the . . . . 255
Apoplexy, on . . . 388
Arm, painful affection of, cured by Acupuncture . . . 31
Arteries, instrument for compressing the Inguinal . . 37
Artificial supports, effects of . 40
Ascites, on . . . 393

Barrenness in prostitutes, cause of 344
Bean, the Carob, on the use of . 171
Black Pepper as a febrifuge . 253
Bladder, irritation of, from stricture of rectum . . . 21
———, numerous calculi in the 343
Bones of the head, alteration in the 434
Brain, hæmatoid tumor in the . 461
Bronchocele, on . . . 389
Butchers, on the malignant pustule of . . . . . 344
Burns and scalds, on the healing of 195

PAGE

Burns, Chloruret of Lime in . 521

Calomel, salivation from a dose of . . . . . 253
Calculi, numerous, in the bladder 343
——— in the Urethra . . 525
——— in the Kidneys, on . 474
Calculus, large, passed from the female Urethra . . 83
——— in the Bladder, on . 477
Cancer, use of Kali Hydriodinici in 82
——— of the Uterus . 250, 479
——— of the Heart . . 517
Cantharides, powdered, case of swallowing . . . 463
Carob Bean, on the use of . 171
Case of Diseased Larynx . . 25
——— painful Affection of the Arm 31
——— Separation of the Foot in a Fœtus . . . . 38
——— Hydatid Ascites . . 114
——— Poisoning from Opium . 115
——— Pulsation of the Veins . 170
——— Tetanus in the Horse . 197
——— Hydrocephalus in a Child . 204
——— very short Funis Umbilicus 205
——— Ossification of the Uterus . 467
——— long-continued Insensibility 370
——— Fistula between the Urethra and Vagina . . 439
Cases of the Plymouth Dock-yard Disease . . . . 175
——— Palsy . . . 252
——— Transfusion in Uterine Hemorrhage . . 273, 380
——— Diseased Spleen . . 277
——— Tic Douloureux . . 281
——— Croup . . . 285
——— Hydrophobia . . 375, 519
Cataract and Gutta Serena, cure of 254
Caustic to destroy the variolous eruption . . 170, 522
Chlor[illegible] of Lime, effects of the 254
Chronic Inflammation of the Larynx and Trachea, on . . 390
Chyle in the veins of the Jejunum 432

PAGE
Clavicle, fracture of, in a fœtus . 254
Clitoris, extirpation of the . 83
Closure of the Pupil in Iritis, on 113
Codeate of Morphia . . 521
Communication by Ulcer between the Bladder and the Rectum, on 477
Complaints of the Head . . 387
Contracted Joints, on the treatment of . . . 215
Contagion of the Yellow Fever, on 249
Croton Oil, on the . . 105
Croup, cases of . . . 285
Cure of a Hydrophobic Patient . 250

Delirium Tremens, on the pathology of . . . . 94
Diabetes, on . . . 475
Diathesis, aneurismal, on the . 433
Diagnostic of Venereal Gonorrhœa 252
Diseased Larynx, case of . . 25
Diseases, on feigned . . 87
——— of Plymouth Dock-yard 175
——— in the Cavity of the Abdomen . . . . 393
——— of the Neck . . 389
——— Chest . . 390
——— Pancreas, on . 472
——— Spleen, on . ib.
Dose of Calomel, salivation from . 253
Dropsy of the Epiploon . . 342
Duration, the mean, of Intermittent Fever . . . 249
Dysentery, on . . . 396

Ear, internal, suppuration of the . 519
Effects of artificial supports . 40
——— the Chloruret of Lime . 254
——— swallowing powdered Cantharides . . . 463
Encysted tumors of the Abdomen 343
Enlargement of the Womb, on . 479
Epilepsy, on . . . 388
Epiploon, dropsy of the . . 342
Ergot of rye, effects of . 1, 100
Euphorbia Lathyris, oil of . 253
Extirpation of the Clitoris . 83
Extra-uterine fœtus . . 255

Feigned diseases, on . . 87
Female urethra, large calculus passed from . . . 83
Fever, Yellow, on the contagion of 249
———, Intermittent, on the mean duration of . . . 249
Fœtus, separation of the foot in a 38
———, fracture of the Clavicle in a 254
———, extra-uterine . . 255
Fumigating Baths, remarks on . . 207
Fumigation, Mercurial, remarks on 12
Funis Umbilicus, very short, case of 205

Gallstones, remarks on . . 471

PAGE
Gonorrhœa, venereal, diagnostic of 252
Gutta Serena and Cataract, cure of 254

Head, alteration in the bones of . 434
Heart, rupture of the . . 433
———, cancer of the . . 517
———, ossification of the . ib.
Hæmatoid tumor in the Brain . 461
Hæmorrhage in the Stomach . 344
——— from the Kidneys, on 475
High operation for the Stone 84, 434
History, natural, of Water . 444
Horse, case of Tetanus in the . 197
Hydatid Ascites, case of . . 114
Hydatids in the Liver, on . 471
——— Kidneys, on . 474
Hydrocephalus, case of, in a Child 204
Hydrophobic case, cure of . 250
Hydrophobia, cases of . 375, 519
Hydrocephalus, on . . 388
Hydrothorax, on . . . 392

Inflammation of the Tongue . 517
——— Peritoneum, on 394
——— Bowels, on . 396
Inflammatory diseases, on the Pathology of . . . . 7
Infants, new-born, morbid alterations in . . . . 81
Injections of Ammonia, in Amenorrhœa . . . . 434
Insensibility, long-continued, case of . . . . 370
Instrument for compressing Arteries 37
Instinct, observations on . . 351
Internal Ear, suppuration of . 519
Intestines, obstructions of, remarks on . . . . 280
Iritis, on closure of the Pupil in . 113

Jejunum, Chyle in the veins of . 432
Joints, contracted, on the treatment of . . . . 215

Kali Hydriodinici, use of, in Cancer 82

Lactucarium, preparation of . 436
Larynx, case of diseased . . 25
Leech, officinal, on the re-production of, in the Antilles . . 255
Lime, effects of the Chloruret of . 254
Lithontriptor, on the . . 348
Lyssæ, on the existence of, in rabid animals . . . . 251

Malignant Pustule of Butchers, on the . . . . 344
Means of curing a Stoop, on the . 210
Mean duration of Intermittent Fever, on the . . . . 249
Mercurial fumigation, remarks on 12
Morphia, Codeate of . . 521

Hooping Cough. Venables on 189

PAGE
Natural history of Water . 444
Nerves, on the structure of . 247

Observations on Pathology . 263
——— Tic Douloureux . 288
——— Instinct . . 351
Obstructions of the Intestines, remarks on . . . 280
Oil of Croton, on the . . 105
—— Euphorbia Lathyris . 253
Ointment of Tartarised Antimony, on the . . . . 346
Opium, on the analysis of . . 111
——, case of poisoning from . 115
Operation for the Stone, case of the high . . . 434
Origin of the Spinal Nerves . 431
Ossification of the Spleen . 252
——— Heart . . 517

Palpitation of the Heart . . 392
Palsy of the Limbs, use of Rhus Toxicodendron in . . 82
——, cases of . . . 252
Pathology of Inflammation, on . 7
——— Delirium Tremens, on 94
———, observations on . 263
Pepper, black, as a febrifuge . 253
Pertussis, on the treatment of . 189
Phthisis Pulmonalis, on . . 391
Plague, remarks on the . . 117
Polypus of the Womb, on . . 478
Powdered Cantharides, effects of swallowing . . . 463
Preparation of Lactucarium . 436
Prostitutes, cause of barrenness in 344
Prussic Acid, on the . . 347
Puerperal Fever, on the use of Turpentine in . . . 33
Pulsation of the Veins, case of . 170
Pustule of Butchers, malignant, on the . . . . 344

Quinine, Sulphate of, on the 253, 437
Quinsy, on the . . . 390

Rabid Animals, existence of Lyssæ in . . . . 251
Rectum, Stricture of, producing Irritation of Bladder . . 21
Red Sulphate of Iron, on the . 254
Removal of a portion of the Scapula 347
Remarks on the Plague . . 117
——— Fumigating Baths . 207
——— Obstructions of the Intestines . . . 280
——— the "Living Skeleton" 282
Reproduction of the Leech in the Antilles . . . 255
Rhus Toxicodendron, use of in Palsy of the Limbs . . 82

PAGE
Rupture of the Heart . . 433

Salivation from a dose of Calomel 253
Scalds and Burns, on the healing of 195
Scapula, on the removal of a portion of the . . . 347
Secretion, diseased, from the Bladder, on . . . . 477
Skeleton, the Living, remarks on . 282
Spinal Nerves, on the origin of . 431
Spleen, ossification of the . 252
——, diseased, case of . . 277
Stone, high operation for . . 84
——, case of high operation for the . . . . 434
Stomach, Hemorrhage of the . 344
——, Softening of the . 433
Stoops, on the means of curing . 210
Structure of the Nerves, on the . 247
Sulphate of Quinine, on the 253, 437
——— Iron, on the red . 254
Supports, artificial, on the effect of 40
Suppuration of the internal Ear . 519

Tetanus in the Horse, case of . 197
Tic Douloureux, observations on the . . . . 288
———, on . . 389
Tongue, Inflammation of the . 517
Transfusion, cases of, in Uterine Hemorrhage . . 273, 380
Treatment of Pertussis, on the . 189
——— contracted Joints, on the . . . . 215
Tubercles in the Liver, on . 470
Tumor, loose, in the region of the Kidney, on a . . . 476
Tumors, encysted, of the Abdomen 343
—— in the Brain, hæmatoid . 461
Turpentine, on the use of in Puerperal Fever . . . 33

Urethra and Vagina, fistula between, case of . . . 439
——, Calculi in the . . 255
Uterus, ossification of, case of . 467
——, Cancer of the . . 250
Uterine Hemorrhage, case of Transfusion in . . 273, 380

Variolous Eruption destroyed by Caustic . . . 170, 522
Veins, Pulsation of the, case of . 170
—— of the Jejunum, Chyle in the 432
Venereal Gonorrhœa, diagnostic of 252

Water, on the Natural History of 444
Wound of the Parietes of the Abdomen, account of . . 466

Yellow Fever, on the contagion of 249

### COLLECTANEA MEDICA.

PAGE

Extracts from Mr. SHAW's "Further Observations on the Lateral or Serpentine Curvature of the Spine" . . . . . 40, 210

Copy of a Dispatch from Lieutenant-General the Right Honourable Sir THOMAS MAITLAND, to the Right Honourable the Earl BATHURST, K.G. dated Corfu, 8th of April, 1819, on the Subject of the Plague . . 117

On the Use of Iodine in Bronchocele; being the Result of Dr. MANSON's Experience in One Hundred and Twenty Cases . . . 297

Extracts from "Lectures and Observations in Medicine." By the late MATTHEW BAILLIE, M.D. . . . . . 386, 469

### CRITICAL ANALYSIS.

### DOMESTIC.

The Elements of Medical Chemistry: embracing only those Branches of Chemical Science which are calculated to illustrate or explain the different Objects of Medicine; and to furnish a Chemical Grammar to the Author's Pharmacologia. Illustrated by numerous Engravings on Wood. By JOHN AYRTON PARIS, M.D. F.R.S. &c. &c. . . . . 45

Observations on the Use of the Colchicum Autumnale in the Treatment of Gout; and on the proper Means of preventing the Recurrence of the Disorder. By CHARLES SCUDAMORE, M.D. F.R.S. &c. . . . 55

An Essay on Egyptian Mummies; with Observations on the Art of Embalming among the ancient Egyptians. By A. B. GRANVILLE, M.D. F.R.S. &c. 127

The Study of Medicine. Second Edition. By JOHN MASON GOOD, M.D. F.R.S. &c. . . . . . . . . . 137

An Essay on Tetanus, founded on Cases and Experiments. By JOSEPH SWAN, Member of the Royal College of Surgeons, and Surgeon to the Lincoln County Hospital . . . . . . . 218

Remarks on Irritative Fever, commonly called the Plymouth Dock-yard Disease; with Mr. DRYDEN's detailed Account of the Fatal Cases, including that of the lamented Surgeon, Dr. BELL. Dedicated, with permission, to Commissioner SHIELD. By JOHN BUTTER, M.D. F.R.S. &c. &c. . . . . . . . . . 229

Medico-Chirurgical Transactions. Vol. XIII. Part I. . 314, 414, 491

Practical Remarks upon Indigestion; particularly as connected with Bilious and Nervous Affections of the Head and other parts; including Observations upon Disorders and Diseases of the Stomach and superior parts of the Alimentary Canal. Illustrated by Cases. By JOHN HOWSHIP, Assistant Surgeon to the St. George's Infirmary, &c. &c. . . 397

A Treatise on the Properties and Medicinal Application of the Vapour-Bath, in its different Varieties, and their Effects in various Species of Diseased Action. By J. GIBNEY, M.D. of the University of Edinburgh, &c. &c. . . . . . . . . . 326

An Essay on the Nature, Causes, and Treatment, of Water in the Brain. By WILLIAM SHEARMAN, M.D. Member of the Royal College of Physicians, &c. . . . . . . . . 481

### FOREIGN.

A Compendious System of Midwifery, chiefly designed to facilitate the Inquiries of those who may be pursuing this Branch of Study. Illustrated by occasional Cases, with fourteen Engravings. By W. P. DEWEES, M.D. Lecturer on Midwifery, Member of the American Phil. Society, &c. 66, 143

PAGE

Tableau des Maladies observées a l'Hotel Dieu, dans les Salles de Clinique de M le Professeur RECAMIER, pendant le premier Trimestre de 1825. Par L. M. MARTINET . . . . . . . . . . 240

De la Membrane Muqueuse Gastro-intestinale, dans l'etat Sans et dans l'etat Inflammatoire ; ou, Recherches d'Anatomie Pathologique sur les divers Aspects, sains et morbids, qui peuvent presenter l'Estomac et les Intestins. Par C. BILLARD, ex-Elève interne des Hopitaux d'Angers, &c. . . 333

Recherches Statistiques sur la Durée moyenne des Fièvres Intermittentes. Memoire lu à l'Institut, par M. BAILLY, de Blois, D.M.P. . . 423

Observations sur l'Hydrophobie; Indices certains pour reconnaître l'existence du Virus Hydrophobique dans un Individu, et Moyens de Prevenir la Developpement de la Maladie, en detruisant le Germe; suivés des principaux ces pratiques, depuis l'année 1813 jusqu'en 1823. Par le Dr. MAROCHETTI, Médecin et Chirurgien à la grande Amirauté de Petersburg, &c. . . . . . . . . . . . 504

### MISCELLANEOUS.

Account of the great Hospital at Munich . . . . . 84
Report of the Associated Apothecaries . . . . . 172
The EDITORS' Account of "the Living Skeleton" . . . . 256

### OBITUARY.

Biographical Sketch of the late JOHN A. SCHETKY, Esq. . . . 258

### MONTHLY CATALOGUE OF BOOKS.

Pages 86, 173, 261, 349, 437, 523.

### METEOROLOGICAL TABLES.

Pages 85, 174, 262, 350, 438, 524.

### NOTICES TO CORRESPONDENTS.

Pages 86, 174, 262, 350, 438, 524.

### ERRATA.

Pages 86, 262.

### PLATES.

Of a Subclavian or Inguinal Tourniquet . . . . 37
Mr. HOBART's Instrument . . . . . . . 439

### NAMES OF AUTHORS,

*Of whose Works, Observations, &c. either a detailed Account, or more or less general View, is given in*

### THE FIFTY-FOURTH VOLUME

### OF THE LONDON MEDICAL AND PHYSICAL JOURNAL.

PAGE

ACHARD, M. on the Reproduction of the officinal Leech in the Antilles . 255
AMUSAT, M. on the Origin of the Spinal Nerves . . . . 431
ANDRAL, M. (fils,) on Encysted Tumors of the Abdomen . . . 343
ASHFORD, Mr. case of Poisoning by Opium . . . . 115

BAILLIE, Dr. Extracts from his unpublished Works . . 386, 469

BAILLY, M. on the mean Duration of Intermittents . . . 249

BASEDOW, Dr. on the Malignant Pustule of Butchers . . . 344

BAUDELOCQUE, M. on Cancer of the Uterus . . . . 250

——————— on an Extra-uterine Fœtus . . . . 255

BLACKETT, Mr. his Instrument for compressing the Inguinal Artery . 37

——————— on the Carob Bean . . . . . . 171

——————— on Tic Douleureux . . . . . . 288

BOGROS, M. on the Structure of the Nerves . . . . 247

BOULU, Dr. on Calculi in the Urethra . . . . . 255

BOYLE, Mr. on the "Living Skeleton" . . . . . 282

BRACCONOT, M. on the Use of the Red Sulphate of Iron . . . 254

BRYER, Dr. on Pulsation of the Veins . . . . . 170

BUSH, Mr. on Scalds and Burns . . . . . . 195

CAVENTOU, M. on the Oil of the Euphorbia Lathyris . . . 253
CHERWIN, Dr. on the Contagion of Yellow Fever . . . . 249
CHOMEL, M. on Ossification of the Heart . . . . . 517
——————— on the Aneurismal Diathesis . . . . . 433
CIVIALE, M. his Lithontriptor . . . . . . 348
COSTA, Dr. on the Non-contagion of Yellow Fever . . . 434

DAVIES, Dr. HENRY, on the Ergot of Rye . . . . 1, 100
DIX, Mr. case of Wound of the Parietes of the Abdomen . . . 466
DOUBLEDAY, Mr. case of Transfusion . . . . . 380
DUVERGIE, M. on Fracture of the Clavicle in a Fœtus . . . 254
——————— on Alteration of the Bones of the Head . . . 434
Dewees Dr. on Midwifery — ← 66. 143.

EGAN, Mr. on Tetanus in the Horse . . . . . 197
ELLIOTSON, Dr. fatal case of Hemorrhage in the Stomach . . . 344
——————— on the Ointment of Tartarised Antimony . . . 346
——————— on the Prussic Acid . . . . . . 347

FERRUS, M. on Softening of the Stomach . . . . . 433
———————, on Rupture of the Heart . . . . . ib.
FOWKES, Mr. case of Ossification of the Uterus . . . . 467
FRANK, Dr. LOUIS, on Pepper as a Febrifuge . . . . 253

PAGE

Gibson, Dr. on Mercurial Fumigation . . . . . . 12

Gibney, Dr. on the Properties and Medical Application of the Vapour Bath 326

Gondret, Dr. on the cure of Cataract and Gutta Serena . . . 254

Good, Dr. Mason, on Instinct . . . . . . . 351

Goodeve, Mr. case of Tracheotemy . . . . . 25

Grafe, Dr. on Kali Hydriodinici in Cancer . . . . 82

——— case of Inflammation of the Tongue . . . . 517

Gray, Mr. Surgical Cases by . . . . . . 204

Green, Mr. remarks on Fumigating Baths . . . . . 207

Gregory, Dr. cases of Croup . . . . . . 285

——— case of Hæmatoid Tumor of the Brain . . . 461

Hartle, Mr. case of Diseased Spleen . . . . . 277

Henry, Mr. sen. on Sulphate of Quinine . . . . . 253

Hobart, Mr. case of Fistula between the Urethra and Vagina . . 439

Hutchison, Mr. Copland, on Feigned Diseases . . . 87

———, case of High Operation for the Stone . . 434

Hutchinson, Mr. on Tic Douloureux . . . . . 281

Janson, M. on the Removal of a great Portion of the Scapula . . 347
Jeston, Mr. on the Analysis of Opium . . . . . 111
Jones, Mr. on the Pathology of Diseases . . . . . 7
——— on the Pathology of Delirium Tremens, Erysipelas, and Cholera 94

Kinneir, Dr. on Oil of Turpentine in Puerperal Fever . . . 33

Langstaff, Mr. on Barrenness in Prostitutes . . . . 344
Legalas, M. on Cancer of the Heart . . . . . 517
Lepine, Mr. case of Hydrophobia . . . . . . 519
Long, Dr. case of Hydatid Ascites . . . . . . 114
Lisfranc, M. on Chloruret of Lime in Burns . . . . 521

Maitland, Sir Thomas, on the Plague . . . . . 117
Mackenzie, Mr. on the Closure of the Pupil in Iritis . . . 113
Manson, Dr. on the Use of Iodine in Bronchocele . . . . 297
Meyer, Dr. on Chyle in the Veins of the Jejunum . . . . 432
Meyraux, M. on Cauterisation of the Pustules of Small-Pox . . 522
Morley, Mr. on Obstructions of the Intestines . . . . 280
Murat, M. case of numerous Calculi in the Bladder . . . 343

Nacquart, M. cases of Palsy . . . . . . . 252

Olivier, Dr. on Dropsy of the Epiploon . . . . . 342
———, M. on the Codeate of Morphia . . . . . 521
Oswald, Mr. case of long-continued Insensibility . . . . 370

Roman, M. on the Preparation of Lactucarium . . . . 436
Rossi, Professor, cure of Hydrophobia . . . . . 250
Ryan, Dr. his Essay on the Natural History of Water . . . 442

Shaw, Mr. on Artificial Supports . . . . . . 40

——— on the Means of curing a Stoop . . . . . 210

Soares, M. on the Existence of Lyssæ in a Mad Dog . . . 251

Sommer, Dr. case of large Calculus in the Female . . . . 83

Schetky, Mr. Biography of . . . . . . . 258

Stoker, Dr. Pathological Observations . . . . . 263

Tegart, Mr. Ed. on the Croton Oil . . . . . . 105
Thomas, Mr. on Salivation from a Dose of Calomel . . . 253
Torrie, Dr. on the Effect of swallowing powdered Cantharides . . 463
Tripe, Mr. on the Plymouth Dock-yard Disease . . . . 175

PAGE
**Velpeau**, Dr. on destroying Variolous Eruption by Caustic . . 170
**Venables**, Dr. case of Pertussis . . . . . . 189
**Veron**, Dr. on Morbid Alterations in new-born Infants . . . 81
**Virey**, M. on the Effects of Chloruret of Lime . . . . 254
**Von Wedekind**, Baron, on the Seat of Gonorrhœa . . . . 252

**Waller**, Mr. case of Transfusion . . . . . . . 273
**Watkinson**, Mr. case of Separation of the Foot in a Fœtus . . 38
**Webster**, Dr. account of a painful Affection of the Arm cured by Acupuncturation . . . . . . . . . . 31
**White**, Mr. cases of Irritation of the Urethra and Bladder . . 21
**Whymper**, Dr. his cases of Hydrophobia . . . . . 375

